# In Memoriam

**Published:** 1886-06-20

**Authors:** 


### IN MEMORIAM.

Mrs. LULA TUCKER HARRIS, daughter of Maj. R. S. Tucker, of Raleigh, N. C., and wife of Dr. Nathan O. Harris, of Atlanta, Ga., was born March 18th, 1861; married April 8th, 1885, and died at Atlanta, Ga., April 23rd, 1886.

One seldom has occasion to briefly chronicle so sad a record as the above; yet, when we who personally knew the subject of this sketch, think of all the peculiar loveliness of her life and character, the pen lingers tenderly over the record, though tears fill the eyes as it is written, and the heart aches at the thought that a woman like this, more of the angel than human, never again on earth will gladden with her sweet and winning presence the eyes and the hearts of those who knew her best and loved her most.

As others have said of Mrs. Harris, so prominent and engaging were her many charms and perfections, it is difficult to single out any especial one. Those who knew her could only feel that they admired and loved her, and that it seemed one could not be in her presence without having a desre to emulate the beauty and purity of her life and character—in a word, to be like her if it were only possible to fashion one's self after the lovely model.

But if I should dwell upon one of her characteristics more than another it would be the utterly unselfish, the pure, tender and loving heart which was manifest in this angelic woman to the greatest degree of any one it has been my good fortune to know. This, I think, was the grand secret of her power to win love and admiration from all with whom she came in contact, and it was the foundation upon which was built all of her brief and beautiful life.

Her peculiarly affectionate and uselfish disposition was shown in a remarkable degree in all her words and acts, not only towards her own parents and relatives, but to the father and mother of her husband: no daughter of their own flesh and blood could have been more filial, submissive and tender. Her husband's friends were her friends. Those he loved were also dear to her; and to the men and women he admired or revered, she gave honor and reverence.

There was one other prominent feature in the character of Mrs. Harris I would like to mention and emphasize, because it is so rarely found among young girls or matrons in our prominent families and representative social circles, and it is this: though reared from infancy in the midst of luxury, culture, and aristocratic surroundings—loved and adored, caressed and complimented to adulation—yet, she "was not spoilt." With grace to adorn the high social position she occupied, yet she had none of the selfishness, jealousy, insincerity, affectation and haughtiness which is getting to be the rule, rather than the exception, in what is called first circles of society. Those who knew her would almost have associated any of these repulsive characteristics with an angel from heaven as with herself. To the immediate relatives of the deceased, especially to our young professional brother, who has been suddenly bereft of such a wife as this—the happy, loving and radiant bride of but a few fleeting months—we can say nothing. His is a sorrow that human consolation cannot assuage, and with a heart wrung with sympathy for hi.n in this overwhelming affliction, we can only commend him to the compassionate ministrations of One who feels for all our woes, and who holds in His tender hands the only balm in Gilead, while we pray that our bereaved brother will, some day, be united in eternal bonds to the angel wife who awaits him on the beautiful shores of the land of the blest. P.

